# Dimer-Dependent Intrinsic/Basal Activity of the Class B G Protein-Coupled Receptor PAC1 Promotes Cellular Anti-Apoptotic Activity through Wnt/β-Catenin Pathways that Are Associated with Dimer Endocytosis

**DOI:** 10.1371/journal.pone.0113913

**Published:** 2014-11-26

**Authors:** Rongjie Yu, Zekai Cui, Mei Li, Yanxu Yang, Jiaping Zhong

**Affiliations:** Institute of Biomedicine, Department of Cell Biology, Jinan University, Guangzhou, China; University of Rouen, France

## Abstract

The high expression of PACAP (pituitary adenylate cyclase-activating polypeptide)-preferring receptor PAC1 is associated with nerve injury and tumors. Our previous report (Yu R, et al. PLoS One 2012; 7: e51811) confirmed the dimerization of PAC1 and found that the M-PAC1 mutation in the N-terminal first Cys/Ala lost the ability to form dimers. In this study, Chinese hamster ovary (CHO-K1) cells overexpressing wild-type PAC1 (PAC1-CHO) had significantly higher anti-apoptotic activities against serum withdrawal-induced apoptosis associated with a lower caspase 3 activity and a higher Bcl-2 level in a ligand-independent manner than those of CHO cells overexpressing the mutant M-PAC1 (M-PAC1-CHO). PAC1-CHO had significantly higher β-catenin, cyclin D1 and c-myc levels corresponding to the Wnt/β-catenin signal than did M-PAC1-CHO. In addition, the Wnt/β-catenin pathway inhibitor XAV939 significantly inhibited the anti-apoptotic activities of PAC1-CHO. Top-flash assays demonstrated that PAC1-CHO had a significantly stronger Wnt/β-catenin signal than did M-PAC1-CHO. Acetylcysteine (NAC) as an inhibitor of the dimerization of PAC1 inhibited the anti-apoptotic activities that were endowed by PAC1 and decreased the Wnt/β-catenin signal in Top-flash assays. In the PAC1 Tet (tetracycline)-on inducible gene expression system by doxycycline (Dox), higher expression levels of PAC1 resulted in higher anti-apoptotic activities that were associated with a stronger Wnt/β-catenin signal. A similar correlation was also found with the down-regulation of PAC1 in the Neuro2a neuroblastoma cell. BiFC combined with fluorescence confocal imaging indicated that during serum-withdrawal-induced apoptosis, PAC1 dimers displayed significant endocytosis. These findings indicate that PAC1 has ligand-independent and dimer-dependent intrinsic/basal activity, conferring cells with anti-apoptotic activities against serum withdrawal, which is involved in the Wnt/β-catenin signal and is associated with the endocytosis of PAC1 dimers. The discovery and study of the dimer-dependent basal activity of PAC1 not only help us understand the physiological and pathological role of PAC1 but also promote the development of drugs targeting PAC1.

## Introduction

PAC1, the neuropeptide pituitary adenylate cyclase-activating polypeptide (PACAP)-preferring receptor, belongs to the class B G protein-coupled receptor (GPCR) family [1],[2]. PACAP is a member of the vasoactive intestinal polypeptide (VIP)/secretin growth hormone/releasing hormone/glucagon superfamily. Except for the PACAP-specific receptor PAC1, which has an affinity for PACAP of approximately 1000-fold higher than that for VIP, PACAP shares two receptors, VPAC1 and VPAC2, with VIP in equal affinity [2]. PAC1 mediates the effects of PACAP in neurotransmitting, neuron-regulating and neuron-protectant functions, such as the inhibition of apoptosis [3] and the regulation of proliferation and differentiation [4]. PAC1 is highly expressed in the central/peripheral nervous system and neuroendocrine organs and tissues, and the elevated expression of PAC1 is associated with several physiological and pathological changes. For example, PAC1 is highly expressed in neuroendocrine tumors, such as gliomas and medulloblastomas [5],[6]. The levels of PAC1 increase significantly in aged rat brains [7], impaired monkey thymuses [8] and degenerative mouse thymuses [9]. The PAC1 genotype is also correlated with chronic stress [10] and post-traumatic stress disorder [11]. The overexpression of the human PAC1 receptor leads to dose-dependent hydrocephalus-related abnormalities in mice [12]. The overexpression levels of PAC1 in several physiological and pathological processes, in our opinion, are closely related to its roles in regulating apoptosis, cell proliferation and differentiation.

The ligand-independent intrinsic/basal activity of GPCRs has been recognized and is considered associated with the basal neural activity of GPCRs *in vivo* [13]. Therefore, it was inferred that PAC1, as a dominant GPCR for neurotransmitter PACAP, may also have ligand-independent intrinsic (also called basal) activity; in other words, PAC1 may be activated independently of the ligand. It was hypothesized that the overexpression of PAC1 would produce ligand-independent basal activity, which would endow the cells with anti-apoptotic activity in a ligand-independent manner. We considered that the confirmation and characterization of the basal activity of PAC1 may help to clarify the role of elevated levels of PAC1 in specific physiological and pathological processes.

The N-terminal first Cys residue of PAC1 is not included in the three conserved cysteines of the extracellular N-terminal domain of class B GPCRs and has not been reported to form any known disulfide bonds. Using bimolecular fluorescence complementation (BiFC) and bioluminescence resonance energy transfer (BRET) assays, our previous research confirmed that this N-terminal first Cys is essential for the dimerization of PAC1; the replacement of this Cys residue with Ala (to produce the mutant M-PAC1) results in failed receptor dimerization (Yu R, Plos One. 2012; 7(12): e51811) [14]. It was found in this research for the first time that Chinese hamster ovary (CHO-K1) cells overexpressing wild-type PAC1 (PAC1-CHO) had a significantly greater anti-apoptotic ability against serum withdrawal than did CHO cells overexpressing the N-terminal first Cys/Ala mutant M-PAC1 (M-PAC1-CHO) in a ligand-independent manner. Furthermore, the cysteine derivative acetylcysteine (NAC), which was confirmed by BRET and BiFC to inhibit the dimerization of PAC1, inhibited the basal activity of PAC1 against serum-withdrawal-induced apoptosis. These results suggest that PAC1 has dimer-dependent basal activity. A related report by Clémence Carron et al. on the dimerization of the frizzled receptor Xfz3, which is sufficient to activate the Wnt/β-catenin pathway [15], prompted us to further explore whether the dimer-dependent basal activity of PAC1 involves the Wnt/β-catenin pathway. Frizzled-3 is a GPCR in mammals that has low but significant sequence similarity to family B GPCRs and shares a similar cysteine-rich domain in the extracellular N-terminus with family B GPCRs [16]. After screening for the effects of several signal pathway inhibitors, we found that the β-catenin pathway inhibitor XAV-939, which is a small-molecule inhibitor of tankyrase 1 (TNKS1), significantly inhibited the anti-apoptotic activity of PAC1-CHO. Moreover, the levels of β-catenin and its target proteins cyclin D1 and c-myc were significantly higher in PAC1-CHO than in M-PAC1-CHO. Moreover, Top-flash assays, as a β-catenin pathway reporter system, were also used to confirm the relationship of the PAC1 basal activity with the Wnt/β-catenin pathway.

The positive correlation of the PAC1 expression level with its basal activity was further determined using a Tet (tetracycline)-on inducible PAC1 expression system with the controlled expression of PAC1 by doxycycline (Dox) and using the down-regulation of PAC1 with shRNA in neuro2a neuroblastoma cells with a naturally high expression of PAC1. Finally, it was found that the significant endocytosis of PAC1 dimers was associated with the basal activity of PAC1 during serum-withdrawal-induced apoptosis.

## Materials and Methods

### Materials and cell lines

All of the materials for the cell culture and transfection reagents were from Invitrogen (Carlsbad, USA). The reagents for the molecular biological techniques were obtained from Takara (Dalian, China) and QIAGEN (Valencia, Spain). The peptide PACAP27 was synthesized by Qiangrao Biological Company (Shanghai, China). A cDNA encoding the mouse PAC1 (Normal/Hop) isoform (a splice variant with no deletion in EC1 and with a hop insertion in the third intracellular cytoplasmic (IC3) loop [17]) was from GeneCopoeia via the Funeng Gene Company (Guangzhou, China). The eukaryotic expression vectors pEYFP (containing the gene encoding yellow fluorescent protein (YFP)), pRluc (containing the gene encoding Renilla luciferase (Rlu)) and pcDNA3.0 were purchased from Yingrun Biological Company (Changsha, China). The pTet-on advanced vector was used to achieve the intrinsic expression of the tetracycline-controlled transcriptional transactivator, and the TRE (Tet-responsive element)-based expression vector pTRE-Tight was used to achieve the Dox-inducible expression of PAC1; both of these vectors were from Clontech Laboratories (Takara, China). Charcoal-stripped fetal bovine serum (CS-FBS), which was used to reduce the interference between the serum and the PAC1 ligands, was from Biological Industries (Biolnd, European). The Caspase 3 Activity Assay Kit and the B-cell lymphoma-2 (Bcl-2) Elisa Assay Kit were from Beyotime Bio-technologic Company (Shanghai, China). The CHO-K1 and neuro2a neuroblastoma cell lines were from the Chinese Academy of Life Sciences (Shanghai, China).

### Plasmids and mutagenesis

The plasmids that were used in this research are listed in Table 1, were constructed as previously described [14] and were confirmed by re-sequencing. In brief, the intact PAC1 gene and the M-PAC1 gene were cloned into the vector pEYFP or pRluc to construct recombinant expression vectors expressing the receptors PAC1-YFP and M-PAC1-YFP that were tagged at the carboxyl terminus with YFP or PAC1-YFP and M-PAC1-YFP tagged at the carboxyl terminus with Rlu. For the BiFC studies, a sequence encoding the 172 N-terminal amino acid residues of YFP with the TAA termination codon that was added by PCR was used to replace YFP to produce the expression vector PAC1-Y/N, which produced a receptor that was tagged at the carboxyl terminus with the 172 N-terminal amino acid residues of YFP. The recombinant vector PAC1-Y/C, which was used to create receptors that were tagged at the carboxyl terminus with the 67 C-terminal amino acid residues of YFP, was constructed in the same way.

**Table 1:** The plasmids information.

Plasmid name	Characters	Usage
pEYFP	Blank vector with YFP	Control in BiFC assay
PAC1-YFP M-PAC1-YFP	Wild type PAC1 and mutant Cys/Ala PAC1 combined with YFP at C-terminus	Receptor overexpression; BRET assay
pRluc	Blank vector with Rlu	Control in Top-flash assay
PAC1-Rlu M-PAC1-Rlu	Wild type PAC1 and mutant Cys/Ala PAC1 combined with Rlu at C-terminus	BRET assay
PAC1-Y/N M-PAC1-Y/N	Wild type PAC1 and mutant Cys/Ala PAC1 combined with the N-terminal fragment of YFP	BiFC assay
PAC1-Y/C M-PAC1-Y/C	Wild type PAC1 and mutant Cys/Ala PAC1 combined with the C-terminal fragment of YFP	
pTRE-Tight-PAC1-YFP	PAC1-YFP cloned into pTRE-Tight	Tet-on inducible expression

For the Tet-on inducible gene expression of PAC1, the DNA fragment encoding PAC1 that was C-terminally tagged with YFP was sub-cloned into the pTRE-Tight vector to construct the recombinant plasmid pTRE-Tight-PAC1-YFP.

### Cell culture and transfection

The CHO cell line CHO-K1, which expresses neither PACAP nor PAC1 [18], was used for the stable expression of the receptor constructs. Western blotting was used to detect PACAP expression in CHO-K1 with a rabbit polyclonal IgG (Santa Cruz Biotechnology, USA) that was raised against the C-terminus of PACAP, while PACAP expression in neuro2a was used as positive control. Then, the CHO cells were grown in Dulbecco's modified Eagle's medium (DMEM) that was supplemented with 10% CS-FBS in a humidified atmosphere of 95% air and 5% CO_2_ at 37°C before transfection. The cells were transfected with vector constructs expressing the wild-type PAC1-YFP or the Cys/Ala mutant M-PAC1-YFP using lipofectamine LTX and Opti-MEM medium (Invitrogen, USA) following the manufacturer's instructions. For stable expression, cells that were transfected with plasmids were selected based on G418 (0.8–1 mg/mL) insensitivity, cloned by successive cycles of limiting dilution and screened by the YFP fluorescence signal. At least three CHO cell clones expressing PAC1-YFP (named PAC1-CHO) or M-PAC1-YFP (named M-PAC1-CHO) permanently at similar receptor levels were used in parallel in the following experiments. The CHO cell clone that was transfected with pcDNA3.1, named pcDNA-CHO, was used as a basal control because the activity of pcDNA-CHO, which does not express PACAP or PAC1, was not correlated with that of PACAP or PAC1. The cells were maintained in DMEM medium that was supplemented with 10% CS-FBS and 0.8 mg/mL G418 with an atmosphere of 95% air and 5% CO_2_ at 37°C. The expression levels of the receptors PAC1-YFP and M-PAC1-YFP were determined by the YFP fluorescence densities in whole-cell lysates using the Victor3 1420 multi-label counter (PerkinElmer) with excitation (460±30 nm) and emission (535±30 nm) filters. Western blotting with a goat polyclonal IgG against the C-terminus of PAC1 that recognizes both rodent and human PAC1 (Santa Cruz Biotechnology, USA) was also used to detect the expression of PAC1-YFP.

### Immunofluorescence

The expression and cell trafficking of PAC1-YFP and M-PAC1-YFP were further determined by immunofluorescence. PAC1-CHO and M-PAC1-CHO cells that were cultured in DMEM with 0.5% CS-FBS at 37°C overnight were fixed with 4% (w/v) paraformaldehyde (PFA) in PBS for a maximum of 5 minutes at room temperature before being washed twice with PBS and incubated in 3% (w/v) BSA in PBS (blocking solution) for one hour at room temperature. For cells that required permeabilization (to permit entry of an antibody that recognizes an intracellular epitope, e.g., BXP-21), a 5-minute incubation in 0.05% (v/v) Triton X-100 in PBS was included before the blocking step. Following blocking, the cells were then incubated with a goat polyclonal IgG against the C-terminus of PAC1 that recognizes both rodent and human PAC1 (1∶100; Santa Cruz Biotechnology, USA) for 1 h at room temperature and then washed twice with PBS. The cells were then incubated for another 1 h with an Alexa 594-conjugated anti-rabbit antiserum (1∶400). After washing with PBS, the cells were viewed using the OLYMPUS converted fluorescence microscope IX71 (Japan) with an excitation of 520±30 nm and an emission of 595±30 nm.

### Bimolecular fluorescence complementation (BiFC)

The BiFC assay is based on the reconstitution of a fluorescent protein molecule upon the re-association of its two non-fluorescent fragments. If YFP is divided into N-terminal (173 amino acid residues) and C-terminal (67 amino acid residues) segments, neither segment exhibits fluorescence when expressed alone. The co-expression of the segments, which are linked to interacting proteins, permits the partial reformation of YFP with the concomitant appearance of the fluorescent signal. For statistical analyses, the CHO cells were seeded equally in a 96-well plate and transfected with the receptor constructs PAC-Y/N+PAC-Y/C and M-PAC-Y/N+M-PAC-Y/C (1.0 µg of DNA per cell divided equally into two plasmids). The fluorescent signals from the cells 48 h after transfection were detected in a Victor3 1420 multi-label counter (PerkinElmer, Wellesley, MA) using excitation (480±30 nm) and emission (535±40 nm) filters. The cells that were transfected with PAC-YFP were used as positive controls, and the cells without transfection were used as negative controls. To examine the effects of the NAC on the dimerization, the transfected CHO cells were incubated with NAC (10 nM) for 2 h at 4°C before collecting the BiFC signals. The experiments were run with at least three replicates in parallel and were repeated three times.

For the BiFC signal observation, the CHO cells were co-transfected with 3 µg of receptor DNA PAC-Y/N+PAC-Y/C or M-PAC-Y/N+M-PAC-Y/C per 10-cm^2^ Petri dish, which was divided equally among the two receptor plasmids. After 48 hours, the cells were observed under an inverted fluorescence microscope using excitation (480±30 nm) and emission (535±25 nm) filters. The trafficking and endocytosis of the PAC1 dimers were imaged using the appropriate spectral settings (excitation, 488 nm argon laser; emission, 545 nm filter; pinhole diameter, 2.3 Airy units) of a confocal microscope (LSM 510 META; Zeiss, USA) that was equipped with a Plan-Apochromat 63×/1.4 numerical aperture oil objective. To detect the endocytosis of PAC1 dimers during serum-withdrawal-induced apoptosis, the live cells in the dish were subjected to serum withdrawal, and fluorescent images were collected at 0, 0.5, and 2 h after serum-withdrawal-induced apoptosis was initiated. Nuclear staining by DAPI was conducted 2 h after the serum withdrawal, and BiFC signals imaged were imaged under a converted fluorescence microscope using excitation (480±30 nm) and emission (535±25) filters.

### Bioluminescence resonance energy transfer assays (BRET)

The CHO cells were seeded equably in a 96-well white OptiPlate and submitted to co-transfection with the receptor construct PAC1-Rluc/PAC1-YFP or M-PAC1-Rluc/M-PAC1-YFP. The BRET assay was initiated 48 hours after transfection by adding the cell-permeant Rlu-specific substrate coelenterazine h to the cell suspension to yield a final concentration of 5 µM in a 96-well white OptiPlate. The BRET signal was collected using a Victor3 1420 multi-label counter (PerkinElmer, Wellesley, MA) with emission filter sets for luminescence (460 nm, bandwidth 25 nm) and fluorescence (535 nm, bandwidth 25 nm). The ratio of fluorescence to luminescence emission from the cells that were transfected with the Rlu-tagged receptor construct alone (1.0 µg of DNA/5×105) was considered background and used to determine the correct factor (Cf = Em535/Em460) that defined the amount of signal in the acceptor portion that was attributable to donor bioluminescence. The BRET ratio was calculated based on the ratio of fluorescence to luminescence emission using the following formula: (Em535–(Em460×Cf))/Em460. For BRET titration (saturation) experiments, the CHO cells were transfected both with a constant amount of donor construct (Rlu-tagged receptor construct at a concentration of 1.0 µg per cell) and with increasing amounts of acceptor construct (YFP-tagged receptor construct at concentrations of 0.3 to 6.0 µg per cell). The BRET ratios were plotted against the acceptor-to-donor ratios. Curves were fit to these data and were evaluated for quality-of-fit based on R2 values using Prism 3.0. When a single-phase exponential curve was found to represent a significantly better fit than the linear function (F test determination with p value <0.05), this curve was used to calculate the BRETmax and BRET50 values. To examine the effects of NAC, the transfected CHO cells were incubated with 10 nM NAC for 2 h at 4°C before the BRET signals were collected. The experiments were run with at least three replicates in parallel and were repeated three times.

### The ligand-dependent activation of PAC1-YFP and M-PAC1-YFP by PACAP

To detect the ligand-dependent activity of PAC1-YFP and M-PAC1-YFP, PAC1-CHO, M-PAC1-CHO and pcDNA-CHO cells with the same cell density (2×10^5^ cells/well) in DMEM with 0.5% CS-FBS were seeded in 96-well plates and incubated overnight at 37°C. The next day, the cells were incubated with or without PACAP at a range of concentrations (1–100 nM) in the absence of CS-FBS for 24 h. The viability of the cells was determined using a colorimetric MTT (methylthiazole tetrazolium bromide) assay (Sigma, USA). Cyclic AMP assays were also used to test the ligand-dependent activity of PAC1 and M-PAC1 against PACAP because an elevation in cAMP levels is a mark of PACAP activation. The PAC1-CHO and M-PAC1-CHO cells were scraped off the surface with a rubber policeman and washed with PBS twice, and the density of the cells was adjusted to 2×10^6^ cells/mL. PACAP was added to a 500-µL cell suspension, and the working concentrations of the peptide varied from 1–100 nM. The reactions were incubated at 37°C for 5 min and were then incubated at room temperature for 20 min after two volumes of 0.2 M HCl was added. The mixture was dissociated by repeated pipetting until the suspension was homogeneous. The precipitate was removed by centrifugation at 1000 *g* for 10 min, the supernatant was collected, and the cAMP quantity was determined using a cAMP ELISA kit (Cayman Chemical, USA). The data were plotted as fold changes in the data from the untreated pcDNA-CHO cells without PACAP (0 nM). The experiments were performed in parallel with at least three replicates and were repeated three times.

### Serum-withdrawal-induced apoptosis

The cells were cultured in CS-FBS to reduce the interference between the serum and PAC1 ligands, such as PACAP and VIP. Serum withdrawal produced ligand-free conditions for the detection of the ligand-independent activity of PAC1. PAC1-CHO, M-PAC1-CHO and pcDNA-CHO cells as well as the Tet-on inducible cells expressing PAC1 at a range of levels (induced with Dox for 48 h) and neuro2a cells were seeded in 6-well plates in DMEM with 10% CS-FBS and were cultured to 80% confluence. The cells were then subjected to serum withdrawal by being cultured with DMEM alone for 48 h with or without the signal inhibitors H-89 (100 µM), XAV-939 (10 µM), and NAC (10 nM). The viability of the remaining cells was determined using the colorimetric MTT assay that is shown below. In addition, the caspase 3 activity and the intracellular levels of the anti-apoptosis factor Bcl-2 were determined following the kit manufacturers' instructions. The pcDNA-CHO cells were used as a basic control. The data are expressed and plotted as fold changes in the levels in pcDNA-CHO. For the Tet-on inducible PAC1 expression cells, the data are expressed and plotted as fold changes in the double-stable Tet-on advanced inducible cells that were treated without Dox (0 ng/mL). The experiments were performed in parallel with at least three replicates and were repeated three times.

### Cell viability assays by MTT

Cell viability was evaluated using the MTT assay, which is based on the reduction of MTT into a blue formazan dye by viable mitochondria. In brief, the medium was discarded from the plates, and the cells were subsequently washed twice with PBS. The cells were then incubated with PBS containing 0.5 mg/mL MTT for 4 h at 37°C in an atmosphere of 5% CO_2_. The solution was removed carefully, and 1 mL of dimethylsulfoxide was added to dissolve the blue-colored formazan particles. The samples were transferred to a 96-well plate, and the absorbance at 570 nm was measured using a Bio-Rad microplate reader (Bio-Rad, USA) with the values expressed in arbitrary units (AU). The experiments were performed in parallel with at least three replicates and were repeated three times. The remaining cell viability was calculated as the percentage of the initial cell viability without serum withdrawal, and the other data are plotted as fold changes in the data from pcDNA-CHO. For the Tet-on inducible expression system, the data are plotted as fold changes in the data from the treatment without Dox.

### Western blotting

To confirm that the Wnt/β-catenin pathway was involved in the ligand-independent intrinsic activity of PAC1, the expression levels of β-catenin, cyclin-D1 and c-myc (two target proteins of β-catenin) were detected by western blotting. In brief, PAC1-CHO, M-PAC1-CHO and pcDNA-CHO cells as well as the Tet-on inducible cells expressing PAC1 at a range of levels (induced with Dox for 48 h) and neuro2a cells were seeded in 6-well plates in DMEM with 10% CS-FBS. The cells were cultured to 80% confluence and were subjected to serum withdrawal by being cultured with DMEM alone with or without the signal inhibitors H-89 (100 µM), XAV-939 (10 µM), and acetylcysteine (10 nM) for 48 h. The cells were lysed in RIPA lysis buffer (Invitrogen, USA), and the cell lysate was subjected to an SDS-PAGE analysis using 4 to 12% Bis-Tris gels (NuPAGE; Invitrogen, USA). After electrophoresis, the proteins were transferred to nitrocellulose membranes that had been incubated in 5% nonfat milk and 0.1% Tween 20/PBS solution at room temperature on a rotating shaker for 2 h to block nonspecific binding sites. The membranes were incubated overnight with antibodies against β-catenin, cyclin D1 and c-myc (Santa Cruz Biotechnology, USA) and were detected using a horseradish peroxidase-linked anti-rabbit IgG secondary antiserum (GE Healthcare, USA). The immunoblots were developed by the application of an enhanced chemiluminescence solution (Pierce Chemical, USA). The bands were analyzed with an imaging densitometer, and the relative protein levels were normalized by the corresponding levels of β-actin. The experiments were performed with at least three replicates and were repeated at least three times.

For the western blotting of PAC1 dimers using a goat polyclonal IgG against the C-terminus of PAC1 that recognizes both rodent and human PAC1 (Santa Cruz Biotechnology, USA), SDS-PAGE was conducted under non-reducing conditions so as not to destroy the dimers of PAC1, while reducing conditions were used in the detection of the expression levels of the receptors.

### The inducible expression of PAC1 with the Tet-on system

The Tet-on advanced inducible gene expression system (Clontech, USA) was used to achieve the controlled expression of PAC1. Target cells that express the Tet-on advanced transactivator and contain an integrated TRE-based expression vector express high levels of the target gene when cultured in the presence of the system's inducer, Dox. First, a Tet-on advanced cell line that intrinsically expressed the Tet-on advanced transactivator was constructed by transfecting CHO cells with the pTet-on advanced vector and selecting positive clones in 0.8–1 mg/mL G418. Second, the recombinant vector pTRE-Tight-PAC1-YFP, accompanied by the linear hygromycin marker (Clontech, USA), was introduced into the positive cells constructed in the first step to create a double-stable Tet-on advanced inducible cell line. Third, the expression of PAC1-YFP in CHO cells was induced by adding Dox (1–100 ng/mL) to the double-stable Tet-on advanced inducible cell clone and incubating for 48 h. The expression of PAC1-YFP was detected by observing YFP fluorescence using an OLYMPUS inverted fluorescence microscope IX71 (Japan) with excitation at 465±30 nm and emission at 525±30 nm. The expression levels of PAC1-YFP were determined by assessing the YFP fluorescence densities in the whole-cell lysate using the Victor3 1420 multi-label counter (PerkinElmer) with excitation (460±30 nm) and emission (535±30 nm) filters. The YFP fluorescence densities (fluorescence/mg protein) were normalized to the protein concentrations of the lysates. Western blotting with a goat polyclonal IgG against the C-terminus of PAC1 that recognizes both rodent and human PAC1 (Santa Cruz Biotechnology, USA) was further used to detect the expression of PAC1-YFP. The double-stable Tet-on advanced inducible cells that were treated with Dox (1–100 ng/mL) or without Dox were further submitted to serum-withdrawal-induced apoptosis. The remaining cell viabilities were assayed by the MTT method, and the caspase 3 activity and Bcl-2 levels were detected following the methods that are mentioned above. The β-catenin, cyclin D1 and c-myc levels, as key proteins that are involved in the Wnt/β-catenin pathway, were detected using western blotting, and the data were normalized by the corresponding levels of the control nucleoporin-p62 and are plotted as fold changes of the cells that were treated without Dox. The experiments were performed with at least three replicates and were repeated at least three times.

### Protein knockdown in neuro2a

Neuro2a neuroblastoma cells were used to detect the effects of down-regulated PAC1 on the PAC1-dimer-dependent basal activities. To knockdown the endogenous PACAP in neuro2a neuroblastoma cells, cells that were seeded in 6-well plates in DMEM with 10% CS-FBS and cultured to 80% confluence were transfected for 6 h with 4 µg per well PACAP shRNA plasmid (Santa Cruz Biotechnology, USA) using lipofectamine LTX and Opti-MEM medium (Invitrogen, USA), after which the cells were washed and incubated with DMEM and 10% CS-FBS for 24 h. Then, puromycin (PM) (10 µg/mL) was added, and the cells were cultured for another 24 h. The cells were harvested for western blot analysis and were probed with a rabbit polyclonal anti-PACAP IgG (Santa Cruz Biotechnology, USA) that was raised against the C-terminus of PACAP and that recognizes both rodent and human PACAP. The cells with endogenous PACAP knockdown were named neuro2a/PACAP^-^ and were submitted to the following assays.

To investigate the effects of the down-regulation of PAC1, the neuro2a/PACAP^-^ cells with endogenous PACAP knockdown that were obtained above were further transfected with three PAC1 shRNA plasmids (Shanghai Genechem Co. Ltd. China) against mouse PAC1 with the following targeted sequences: GAATCCACTACACAGTATT, CACTATTCGGAATCCACTA and TACGCTGAGACTCTACTTT. The cells were seeded in 96-well plates in DMEM with 10% CS-FBS and 10 µg/mL PM, cultured to 80% confluence and transfected for 6 h with 250 ng per well PAC1 shRNA plasmids (equally divided into three plasmids) or 250 ng per well control plasmids (Shanghai Genechem Co., Ltd. China) using lipofectamine LTX and Opti-MEM medium (Invitrogen, USA), after which the cells were washed and incubated with DMEM and 10% CS-FBS for 24 h. Then, the cells were harvested for western blot analysis with a goat polyclonal IgG against the C-terminus of PAC1 that recognizes both rodent and human PAC1 (Santa Cruz Biotechnology, USA).

Neuro2a/PACAP^-^ cells that were transfected with PAC1 shRNA plasmids (+) or control plasmids (-) were further submitted to serum-withdrawal-induced apoptosis following the procedure that is described above. The remaining cell viabilities and the protein levels of β-catenin, cyclin D1 and c-myc were assayed using MTT and western blotting following the methods that are described above. The data are plotted as the fold changes in the cells that were transfected with control plasmids (-). The experiments were performed with at least three replicates and were repeated at least three times.

### TOP-flash assay

The β-catenin reporter plasmid, TOP-flash, and its mutant control, FOP-flash, were purchased from Millipore Corporation. PAC1-CHO, M-PAC1-CHO and pcDNA-CHO cells were seeded onto 24-well plates in DMEM with 10% CS-FBS and cultured to 80% confluence, after which each cell line was submitted to transfection with Top-flash or FOP-flash at 1 µg/well and the pRluc *Renilla* luciferase plasmid (0.1 µg/well) used as an internal control for transfection efficiency. After the cells were cultured in DMEM and 10% CS-FBS for 24 h after transfection, they were submitted to serum-withdrawal-induced apoptosis with or without the signal inhibitors H-89 (100 µM), XAV-939 (10 µM), and acetylcysteine (10 nM) for another 24 h. Then, the cells were lysed, and the luciferase activities were measured using a Dual-Glo Luciferase Assay System (Promega, USA) in a PerkinElmer Victor3 1420 multi-label counter (Wellesley, MA). The relative luciferase activities are expressed as the ratio of TOP-flash/FOP-flash luciferase activity, and the data were plotted as fold changes in the data from the pcDNA-CHO cells. The experiments were performed with at least three replicates and were repeated at least three times.

For the PAC1 Tet-on inducible expression system, the double-stable Tet-on advanced inducible cells were plated onto 24-well plates in DMEM with 10% CS-FBS and cultured to 80% confluence, after which the cells were submitted to transfection with Top-flash (1 µg/well) +pRluc (0.1 µg/well) and Fop-flash (1 µg/well) +pRluc (0.1 µg/well). After the cells recovered from transfection in DMEM and 10% CS-FBS for 24 h, they were submitted to serum-withdrawal-induced apoptosis with Dox (1–100 ng/mL) or without Dox for another 24 h. Then, the cells were lysed, and the luciferase activities were measured using a Dual-Glo Luciferase Assay System (Promega, USA) in a PerkinElmer Victor3 1420 multi-label counter (Wellesley, MA). The relative luciferase activities are expressed as the ratio of TOP-flash/FOP-flash luciferase activity, and the data are plotted as fold changes in the data from the cells that were treated without Dox (0 ng/mL). The experiments were performed with at least three replicates and were repeated at least three times.

### Statistical analysis

The statistical analysis was performed with GraphPad Prism using an unpaired t-test. The results were expressed as the mean ± S.E. (standard error). Differences with p<0.01 were considered statistically significant.

## Results

### NAC is an inhibitor of the dimerization of PAC1

**Figure 1:**
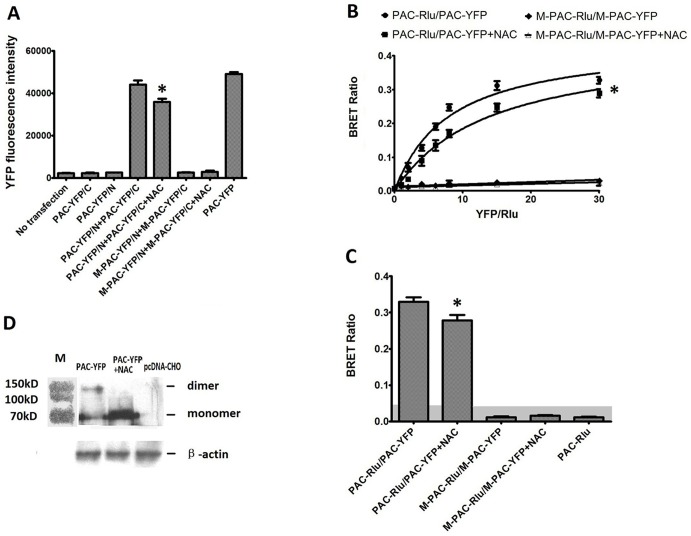
Effects of the NAC on the dimerization of PAC1. (A) BiFC assays. Shown were YFP fluorescence intensity re-produced by the transfection of the receptor constructs as indicated. The cells without transfection were used as negative control and the cells transfected with PAC-YFP as positive control. Exogenous NAC (10 nM) decreased the YFP fluorescence intensity produced by PAC-Y/N+PAC-Y/C significantly (*, P<0.01 PAC-Y/N+PAC-Y/C+NAC vs. PAC-Y/N+PAC-Y/C), while the transfection of M+PAC-Y/N+M+PAC-Y/C produced no YFP fluorescence signals. Data were presented as means ± S.E. of three independent experiments. (B) Saturation BRET. Shown were the BRET saturation curves plotted as a ratio of YFP fluorescence to Rlu luminescence that were observed for tagged receptor constructs studied with a fixed amount of donor and increasing amounts of acceptor. PAC-Rluc/PAC-YFP receptor constructs yielded exponential curves that reached asymptotes indicating significant homo-dimerization of PAC1, while M-PAC-Rluc/M-PAC-YFP yielded curves not different from a straight line, indicating that D-PAC1 lost the ability to form dimers. The addition with NAC (10 nM) at 2 h before the BRET signal assay lowered the curves significantly (*, P<0.01 PAC-Rluc/PAC-YFP+NAC vs. PAC-Rluc/PAC-YFP). The data were represented as the means ± S.E. of three independent experiments. (C) Static BRET. BRET ratios for CHO cells expressing receptor constructs as indicated. For static BRET, a total of 1.0 µg of DNA per well divided equally among the noted constructs in each condition was utilized. The shaded area represents the nonspecific BRET signal generated between PAC-Rlu and soluble YFP protein, with BRET signals above this area considered to be significant. As shown the BRET ratio in PAC-Rluc/PAC-YFP CHO cells incubated with NAC (10 nM) was significantly lower than that in cells without treatment with NAC (*, P<0.01 PAC-Rluc/PAC-YFP+NAC vs. PAC-Rluc/PAC-YFP). The data were presented as the means ± S.E. of three independent experiments. (D) Western blotting analysis with a goat polyclonal IgG against the C-terminus of PAC1 using non-reductive SDS-PAGE. When PAC-YFP expressing cells incubated with exogenous NAC (10 nM), as shown, the band with the molecular weight (about 160 kD) consistent with the molecular weight of the PAC1 dimer was weakened by the presence of NAC (10 nM). All these results showed that NAC was an inhibitor of the dimerization of PAC1, which offered us a tool to analysis the relation of the dimerization of PAC1 with its basal activity.

As shown in Fig. 1, M-PAC1 did not exhibit significant BiFC and BRET signals, confirming that M-PAC1 lost the ability to form dimers, which is consistent with our previous report [14].

NAC, as a derivative of cysteine, was hypothesized to inhibit the dimerization of PAC1 because a previous mutation study confirmed that the N-terminal Cys is essential for the dimerization of PAC1 [14]. BiFC, BRET and western blotting were used to detect the effects of NAC (10 nM) on the dimerization of PAC1. The transfected CHO cells were incubated with 10 nM NAC for 2 h at 4°C before the BiFC and BRET signals were collected and western blotting was performed. As shown in Fig. 1A, the statistical analysis of BiFC showed that NAC (10 nM) significantly decreased the YFP fluorescence intensity that was produced by PAC-Y/N+PAC-Y/C (P<0.01, PAC-Y/N+PAC-Y/C+NAC vs. PAC-Y/N+PAC-Y/C). Both static and saturation BRET (Fig. 1B, C) showed that NAC (10 nM) significantly decreased the BRET ratio that was produced by PAC1 dimerization (P<0.01, PAC-Rluc/PAC-YFP+NAC vs. PAC-Rluc/PAC-YFP). The results of western blotting (Fig. 1D) showed that the band with the molecular weight of approximately 160 kD, which is consistent with the molecular weight of the dimer, was significantly weakened in the cells that were incubated with NAC (10 nM). These results indicate that NAC (10 nM) inhibited the dimerization of PAC1 and that NAC (10 nM) can be used as a tool to detect the relation of PAC1 dimerization with its basal activity.

### PAC1 had less ligand-dependent activity than M-PAC1

**Figure 2:**
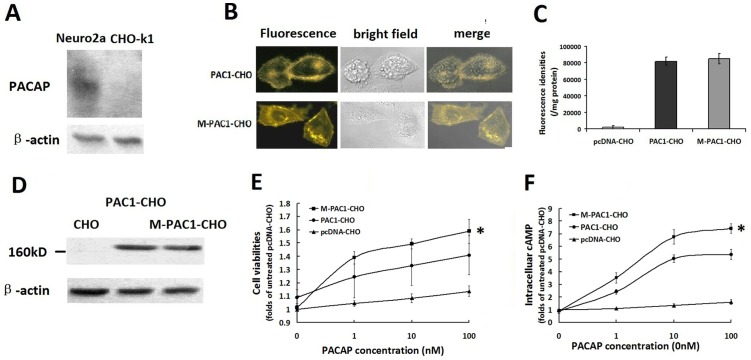
The ligand-dependent activation of PAC1 and M-PAC1 by PACAP. (A) Western blotting of endogenous PACAP in CHO-K1 and neuro2a cells. As shown CHO-K1 had no detectable endogenous PACAP, while neuro2a cells produced endogenous PACAP. (B) The expression of PAC1-YFP and M-PAC1-YFP detected by immunofluorescence. Shown were immunofluorescence results of PAC1-CHO and M-PAC1-CHO cells cultured in DMEM with 0.5% CS-FBS at 37°C overnight, which indicated that both PAC1 and M-PAC1 trafficked normally to the plasma membrane and 0.5% CS-FBS induced no significant receptors endocytosis. (C) Fluorescence densities assays. Shown were the YFP fluorescence densities in the whole cell lysate detected using the Victor3 1420 multi-label counter with excitation (460±30 nm) and emission (535±30 nm), indicating that the expression levels of PAC1-YFP in CHO cells were equal to those of M-PAC1-YFP. (D) Western blotting assays using reductive SDS-PAGE. Western blotting with a goat polyclonal IgG against the C-terminus of PAC1 in the reductive condition showed that there were similar bands with the molecular weight about 160 kD in PAC1-CHO and M-PAC1-CHO, but not in CHO. (E) The cell viabilities of PAC1-CHO and M-PAC1-CHO cells promoted by PACAP. The data were plotted as the fold changes of the treatment without PACAP (0 nM). After the cells were submitted the addition of PACAP (1–100 nM) in the absence of CS-FBS for 24 h, MTT assays showed that PACAP exerted more significant proliferative effects on M-PAC1-CHO than on PAC1-CHO (*, P<0.01, M-PAC1-CHO vs. PAC1-CHO), indicating that the activation level of PAC1 by PACAP was lower than that of M-PAC1. (F) The intracellular cAMP levels induced by PACAP (1–100 nM) in PAC1-CHO and M-PAC1-CHO cells. After the data were plotted as the fold changes of the treatment with 0 nM PACAP, it was shown that the intracellular cAMP levels in M-PAC1-CHO cells induced by PACAP were significantly higher than the intracellular cAMP levels in PAC1-CHO cells induced by PACAP (*, P<0.01, M-PAC1-CHO vs. PAC1-CHO). The data were represented as the means ± S.E. of three independent experiments.

Western blotting was used to detect the expression of endogenous PACAP in two cell lines: CHO-K1 cells and neuro2a cells. As shown in Fig. 2 A, CHO-K1 did not express endogenous PACAP, which is consistent with the previous report by Okazaki et al. [18], while neuro2a cells produced endogenous PACAP. CHO-K1 cells were further used to detect the ligand-independent basal activity of PAC1.

Immunofluorescence, fluorescence density and western blotting were used to detect the expression of PAC1-YFP and M-PAC1-YFP in CHO cells (Fig. 2 B, C, D). As shown in Fig. 2 B, both PAC1-YFP and the N-terminal Cys/Ala mutant M-PAC1-YFP were trafficked to the plasma membrane normally in 0.5% CS-FBS overnight, and there was no significant difference in the receptor locations between PAC1-YFP and M-PAC1-YFP. The fluorescence density assay and the western blotting assay in the total cell lysate both demonstrated that the expression level of PAC1-YFP in the PAC1-CHO cells was equal to the expression level of M-PAC1-YFP in the M-PAC1-CHO cells (Fig. 2 C, D).

When the activation specificities of PAC1-YFP and M-PAC1-YFP by PACAP (1–100 nM) were compared, it was found that the proliferation of PAC1-CHO that was induced by PACAP was significantly weaker than the proliferation of M-PAC1-CHO that was induced by PACAP (Fig. 2 E, P<0.01, M-PAC1-CHO vs. PAC1-CHO) and that the intracellular cAMP levels that were induced by PACAP in PAC1-CHO were significantly lower than those in M-PAC1-CHO that were induced by PACAP (Fig. 2 F, P<0.01, M-PAC1-CHO vs. PAC1-CHO). These results indicate that PAC1-YFP has a weaker sensitivity to PACAP than does M-PAC1-YFP. As previously demonstrated, wild-type PAC1 and the mutant M-PAC1 were trafficked to plasma membranes normally, but PAC1 forms dimers while M-PAC1 cannot. Therefore, it was suggested that the PAC1 dimers on the plasma membrane might interfere with the binding of PACAP to the PAC1 monomer, causing the M-PAC1 monomer to have a higher ligand-dependent activity than that of the PAC1 dimer.

### The ligand-independent activity of PAC1 is involved in the Wnt/β-catenin pathway and is dimer dependent

**Figure 3:**
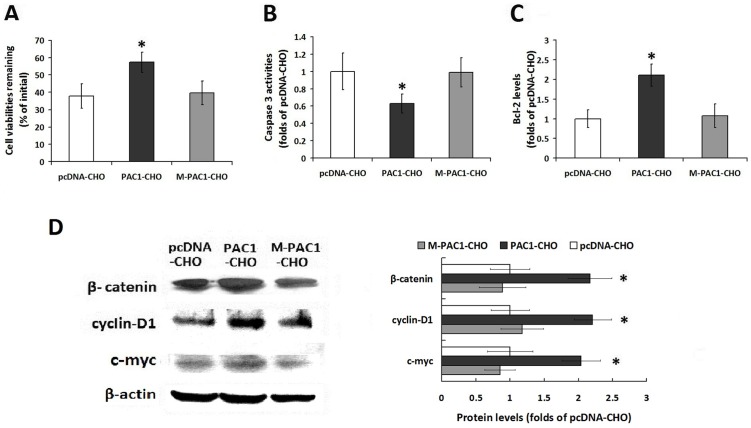
The ligand independent activity of PAC1 and M-PAC1 against serum withdrawal induced apoptosis. (A) The remaining cell viabilities of PAC1-CHO, M-PAC1-CHO and pcDNA-CHO cells 48 h after serum withdrawal. When the data were plotted as the percentage of the initial cell viability without serum withdrawal, it was shown that PAC1-CHO had remaining cell viability (57.34±5.91%) that was significantly higher than that of M-PAC1-CHO (36.96±6.85%) or pcDNA-CHO (37.89±7.11%) (*, P<0.01, PAC1-CHO vs. pcDNA-CHO and M-PAC1-CHO). (B) The intracellular caspase3 activities after serum withdrawal. The reactions of pcDNA-CHO were considered not result from PAC1 because pcDNA-CHO did not express PAC1 or PACAP; therefore, all the data were plotted as fold changes in pcDNA-CHO. As shown, PAC1-CHO had significantly lower caspase3 activity than M-PAC1-CHO or pcDNA-CHO (*, P<0.01, PAC1-CHO vs. pcDNA-CHO and M-PAC1-CHO), whereas there was no significant difference between M-PAC1-CHO and pcDNA-CHO. (C) The intracellular Bcl-2 levels after serum withdrawal. After the data were plotted as the fold changes of pcDNA-CHO, it was shown that PAC1-CHO had significantly higher Bcl-2 level about 2 folds of that in M-PAC1-CHO or pcDNA-CHO (*, P<0.01, PAC1-CHO vs. pcDNA-CHO and M-PAC1-CHO). The data were represented as the means ± S.E. of three independent experiments. (D) The detection of β-catenin, cyclin D1 and c-myc levels in PAC1-CHO, M-PAC1-CHO and pcDNA-CHO cells by western blotting. The western blotting results and the statistical analysis showed that the levels of β-catenin, cyclin D1 and c-myc (tow targets of β-catenin) in PAC1-CHO cells were significantly higher than those in M-PAC1-CHO or pcDNA-CHO cells (*, P<0.01, PAC1-CHO vs. pcDNA-CHO and M-PAC1-CHO). These findings indicated that overexpression of wild type PAC1 endowed CHO with anti-apoptotic activities against serum withdrawal, suggesting that PAC1 had ligand independent basal activity, while M-PAC1 did not. And Wnt/β-catenin signals were involved in the anti-apoptotic activity of PAC1-CHO. The data were represented as the means ± S.E. of three independent experiments.

Serum-withdrawal-induced apoptosis in the PAC1-CHO, M-PAC1-CHO and pcDNA-CHO cells (the cells that were transfected with pcDNA) was used to detect the ligand-independent activity of wild-type PAC1 and its mutant M-PAC1; pcDNA-CHO was used as a basic control. After withdrawing serum for 48 h, the remaining viability of the PAC1-CHO cells (57.34±5.91%) was significantly higher than that of the M-PAC1-CHO cells (36.96±6.85%) and the pcDNA-CHO cells (37.89±7.11%) (Fig. 3 A, P<0.01, PAC1-CHO vs. pcDNA-CHO and M-PAC1-CHO), whereas there was no significant difference between M-PAC1-CHO and pcDNA-CHO. When the data were plotted as fold changes in the data in pcDNA-CHO, which did not express PACAP nor PAC1, the results showed that the M-PAC1-CHO cells had caspase 3 activity and Bcl-2 levels that were equal to those of pcDNA-CHO, whereas PAC1-CHO had significantly lower caspase 3 activity and significantly higher Bcl-2 levels than those of M-PAC1-CHO and pcDNA-CHO (Fig. 3 B, C; P<0.01, PAC1-CHO vs. M-PAC1-CHO and pcDNA-CHO).

In our opinion, the withdrawal of serum prevented interference from any ligands, such as PACAP or VIP, in the serum; furthermore, as shown above, the ligand-dependent activity of PAC1 was weaker than the ligand-dependent activity of M-PAC1. Therefore, it was deduced that PAC1 had intrinsic/basal activity, which conferred anti-apoptotic activities to the PAC1-CHO cells in a ligand-independent manner.

To verify the hypothesis that the Wnt/β-catenin pathway is involved in the basal activity of PAC1, the Wnt/β-catenin-pathway-related proteins, including β-catenin, cyclin-D1 and c-myc (two targets of β-catenin), were detected in PAC1-CHO, M-PAC1-CHO and pcDNA-CHO cells using western blotting. After the data were plotted as fold changes in the data from pcDNA-CHO, it was discovered that PAC1-CHO had significantly higher levels of β-catenin, cyclin-D1 and c-myc than those of pcDNA-CHO or M-PAC1-CHO (Fig. 3 D, P<0.01, PAC1-CHO vs. pcDNA-CHO and M-PAC1-CHO). These results indicate that the ligand-independent basal activity of PAC1 involves the Wnt/β-catenin pathway.

**Figure 4:**
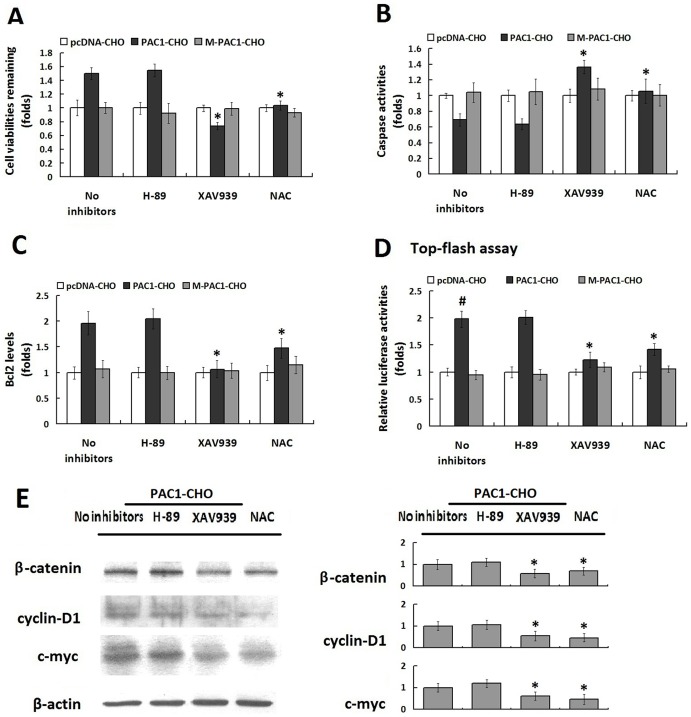
The basal activity of PAC is Wnt/β-catenin pathway involved and dimer dependent. (A) The effects of the cell signaling inhibitors H-89, XAV939 and PAC1 dimerization inhibitor NAC on the remaining cell viabilities of PAC1-CHO, M-PAC1-CHO and pcDNA-CHO 24 h after serum withdrawal. All the data were plotted as the fold changes in the data of pcDNA-CHO because the changes in pcDNA-CHO had no correlation with PAC1. It was shown that the β-catenin signal inhibitor XAV939 (10 µM) and PAC1 dimerization inhibitor NAC (10 nM) significantly decreased the viabilities of PAC1-CHO cells (*, P<0.01, PAC1-CHO/XAV939 and PAC1-CHO/NAC vs. PAC1-CHO/no inhibitors), whereas the PKA inhibitor H-89 (100 µM) did not exert significant effect on the cells viability. The effects of H-89, XAV939 and NAC on the caspase3 activities (B) and Bcl-2 levels (C) in PAC1-CHO, M-PAC1-CHO and pcDNA-CHO 24 h after serum withdrawal showed that XAV939 (10 µM) and NAC (10 nM) exerted significant inhibitory effects on the anti-apoptotic activity of PAC1-CHO by increasing the caspase3 activity and decreasing the Bcl-2 level in the PAC1-CHO cells significantly (*, P<0.01, PAC1-CHO/XAV939 and PAC1-CHO/NAC vs. PAC1-CHO/no inhibitors), whereas H-89 (100 µM) did not have the similar inhibitory effects. (D)Top-flash assays. PAC1-CHO, M-PAC1-CHO and pcDNA-CHO cells were transfected with Top-flash + pRluc and Fop-flash + pRluc respectively. pRluc was used here as the internal control for transfection efficiency. After the transfection, cells were submitted to serum-withdraw induced apoptosis with or without the signal inhibitors H-89 (100 µM), XAV-939 (10 µM), and acetylcysteine (10 nM) for 24 h. And then cells were lysed and luciferase activities were measured. Relative luciferase activities were expressed as the ratio of TOP-flash/FOP-flash luciferase activity and the data were plotted as fold changes in the data from pcDNA-CHO cells. It was found that without inhibitors the relative luciferase activity in PAC1-CHO cells was significantly higher than that in M-PAC1-CHO and pcDNA-CHO (#, P<0.01, PAC1-CHO/no inhibitors vs. M-PAC1-CHO/no inhibitors and pcDNA-CHO/no inhibitors) after serum withdrawal. The addition of Wnt/β-catenin signal inhibitor XAV939 (10 µM) and PAC1 dimerization inhibitor NAC (10 nM) significantly decreased the relative luciferase activity in PAC1-CHO (*, P<0.01, PAC1-CHO/XAV939 and PAC1-CHO/NAC vs. PAC1-CHO/no inhibitors), indicating that the basal activity of PAC1 was Wnt/β-catenin involved and dimer dependent. The data were represented as the means ± S.E. of three independent experiments. (D) Western blotting showed XAV939 (10 µM) and NAC (10 nM) significantly decreased the β-catenin, cyclin D1 and c-myc levels in PAC1-CHO cells. (*, P<0.01, PAC1-CHO/XAV939 and PAC1-CHO/NAC vs. PAC1-CHO/no inhibitors), whereas H-89 (100 µM) did not have the similar inhibitory effects. The data were represented as the means ± S.E. of three independent experiments.

Some cell-signal inhibitors, including the PKA inhibitor H89 and the β-catenin signal inhibitor XAV939, were used to confirm that the basal activity of PAC1 is involved in the Wnt/β-catenin pathway. NAC, which was demonstrated above to be an inhibitor of PAC1 dimerization, was used to determine whether the basal activity of PAC1 was dimer-dependent. XAV939 (10 µM) and NAC (10 nM) significantly inhibited the basal activity of PAC1 by decreasing the remaining cell viabilities, reducing the intracellular Bcl-2 levels and promoting caspase 3 activities (Fig. 4 A, B, C, P<0.01, PAC1-CHO/XAV939 and PAC1-CHO/NAC vs. PAC1-CHO/no inhibitors). Moreover, when the Wnt/β-catenin signal was detected using the Top-flash assay, the results showed that after the luciferase activities were plotted as the fold changes in pRluc-CHO, the relative luciferase activity in PAC1-CHO cells was almost 2-fold that in M-PAC1-CHO and pRluc-CHO after serum withdrawal (Fig. 4 D, P<0.01, PAC1-CHO/no inhibitors vs. M-PAC1-CHO/no inhibitors and pRluc-CHO/no inhibitors). The addition of XAV939 (10 µM) and NAC (10 nM) before serum withdrawal significantly inhibited the relative luciferase activity in PAC1-CHO cells (Fig. 4 D, P<0.01, PAC1-CHO/XAV939 and PAC1-CHO/NAC vs. PAC1-CHO/no inhibitors). Western blotting assays (Fig. 4 E) showed that the levels of β-catenin, cyclin D1 and c-myc in PAC1-CHO cells decreased by the addition of XAV939 (10 µM) and NAC (10 nM) significanltly. These results indicate that the basal activity of PAC1 conferring cells with anti-apoptotic activities against serum withdrawal involved the Wnt/β-catenin pathway and was dimer dependent.

These data demonstrate that the basal activity of PAC1 in a ligand-independent manner endowed cells with increased remaining cell viabilities by promoting anti-apoptotic abilities against serum withdrawal-induced apoptosis and that the basal activity of PAC1 involved the Wnt/β-catenin pathway and depended on the dimerization of PAC1.

### The correlation between PAC1 levels and its basal activity

**Figure 5:**
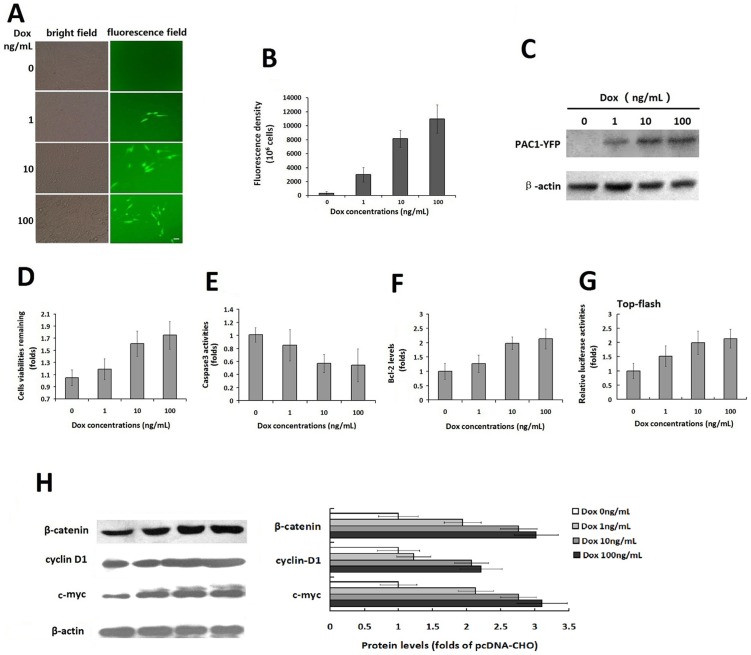
The correlation of the PAC1 levels with its basal activity in Tet-on inducible expression system. The fluorescence microscopic observation (A) and the fluorescence density assays (B) of the PAC1-YFP expression induced by Dox (0–100 ng/mL) in Tet-on inducible system. The fluorescence microscopic images showed that the numbers of cells with YFP fluorescence increased following increases in the concentration of Dox (1–100 ng/mL), whereas there was no fluorescence observed without induction by Dox (0 ng/mL). Bar, 20 µm. The YFP fluorescence densities, assayed using the Victor3 1420 multi-label counter, increased with the concentration of Dox (1–100 ng/mL), indicating that the expression levels of PAC1 were controlled by Dox in a concentration-dependent manner. (C) Western blotting of the inducible expression of PAC1-YFP. The western blotting with goat polyclonal IgG against the C-terminus of PAC1 using reductive SDS-PAGE showed the bands corresponding to PAC1-YFP deepened with the increase of Dox (1–100 ng/mL), while no band corresponding to PAC1-YFP was found in the treatment without Dox (0 ng/mL). The remaining cell viabilities (D), the caspase3 activity (E) and the Bcl-2 levels (F) after serum withdrawal in the double-stable Tet-on advanced inducible cells treated with Dox (1–100 ng/mL) were plotted as the fold changes in the data from the cells treated without Dox (0 ng/mL). It was shown that the higher concentrations of Dox induced higher expression levels of PAC1-YFP, which in turn led to the higher anti-apoptotic activity of the cells, including higher remaining cell viability, lower caspase3 activity and higher Bcl-2 level. (G) Top-flash assays. In double-stable Tet-on advanced inducible cells, after the transfection with Top-flash + pRluc or Fop-flash + pRluc, cells were submitted to serum-withdraw induced apoptosis with Dox (1–100 ng/mL) or without Dox for another 24 h. And then cells were lysed and luciferase activities were measured. Relative luciferase activities were expressed as the ratio of TOP-flash/FOP-flash luciferase activity and the data were plotted as fold changes in the data from the cells treated without Dox (0 ng/mL). It was shown that the relative luciferase activities increased following the increase of Dox (1–100 ng/mL), indicating that the higher expression levels of PAC1-YFP induced by higher concentration of Dox resulted into stronger Wnt/β-catenin signals. (H) Western blotting of β-catenin, cyclin D1 and c-myc corresponding to Wnt/β-catenin pathway. After the protein expression levels were normalized by the corresponding levels of the control nucleoporin-p62 and plotted as the fold changes of the cells treated without Dox, it was shown that in the cells expressing a range of PAC1-YFP induced by Dox (0–100 ng/mL), β-catenin, cyclin D1 and c-myc levels increased following the increases of the PAC1 levels. All these data suggested the significant positive correlation of the PAC1 levels with the anti-apoptotic activities involved with Wnt/β-catenin signals. The data were represented as the means ± S.E. of three independent experiments.

To verify the ligand-independent basal activity of PAC1, the correlation of the level of PAC1 with its basal activity was assessed. After the double-stable Tet-on advanced inducible cell line was constructed, Dox at a range of concentrations (1–100 ng/mL) was added to induce the expression of PAC1-YFP, which was detected using fluorescence microscopy, fluorometry and western blotting (Fig. 5 A, B, C). Dox (1–100 ng/mL) induced the significant concentration-dependent expression of PAC1-YFP. Using this Dox-dependent PAC1 expression model, the activity of the cells with a range of PAC1 levels against serum-withdrawal-induced apoptosis was assessed (Fig. 5 D, E, F). After the data were plotted as fold changes in the treatment without Dox (0 ng/mL), it was shown that the remaining cell viabilities increased 48 h after serum withdrawal following the increase of the expression level of PAC1 (Fig. 5 D). The caspase 3 activity and the Bcl-2 level were positively correlated with the PAC1 level (Fig. 5 E, F). Top-flash assays (Fig. 5G ) in the PAC1 Tet-on inducible expression system showed that the relative luciferase activities presenting the Wnt/β-catenin signals increased following the increased PAC1 levels. Furthermore, western blotting assays (Fig. 5 H) showed that the levels of β-catenin, cyclin D1 and c-myc corresponding to the Wnt/β-catenin pathway also increased following the increased PAC1 levels. These results indicate that higher PAC1 expression resulted in higher anti-apoptotic activity of the cells with higher levels of Wnt/β-catenin signaling.

**Figure 6:**
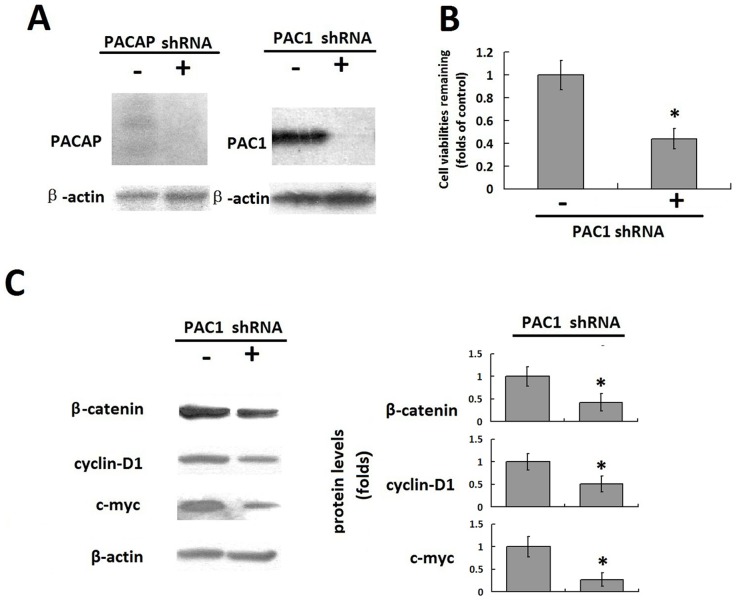
The correlation of PAC1 knockdown with its basal activity in Neuro2a. (A) Knockdown of endogenous PACAP and PAC1 with shRNA in Neuro2a. Western blotting assays showed that shRNA against PACAP significantly diminished the expression of endogenous PACAP in neuro2a/PACAP^-^, and further transfection with shRNA plasmids against PAC1 (+) to neuro2a/PACAP^-^ cells decreased the PAC1 levels significantly, while control plasmids (-) did not interfere with expression of PAC1. The knockdown of PACAP and PAC1 in neuro2a produced a chance for the detection of the correlation of PAC1 down-regulation with its ligand independent basal activity. (B) The remaining cell viabilities of nero2a/PACAP^-^ transfected with PAC1 shRNA plasmids (+) or control plasmid (-). After the data were plotted as the fold changes in the cells transfected with control plasmids (-), it was shown that down-regulation of PAC1 with PAC1 shRNA plasmids (+) decreased the remaining cell viabilities to almost a half of the remaining cell viabilities transfected with control plasmids (-) 48 h after serum withdrawal (*, P<0.01, shRNA + vs. shRNA-). (C) Western blotting of β-catenin, cyclin D1 and c-myc in the nero2a/PACAP^-^ cells transfected with PAC1 shRNA plasmids (+) or control plasmids (-). After the relative protein levels were normalized by the corresponding levels of the control nucleoporin-p62 and plotted as the fold changes in the cells transfected with control plasmids (-), it was shown that PAC1 shRNA plasmids (+) significantly decreased the levels of β-catenin, cyclin D1 and c-myc compared with control plasmids (+)(*, P<0.01, shRNA+ vs. shRNA-). These data suggested that down-regulation of PAC1 in the natural cells such neuro2a with high expression of PAC1 inhibited the anti-apoptotic activities in the ligand free condition. The data were represented as the means ± S.E. of three independent experiments.

Neuro2a cells with a naturally high expression of PAC1 were also used to detect the correlation of the PAC1 level with its basal activity. First, the endogenous PACAP was knocked down by shRNA against PACAP to produce neuro2a/PACAP^-^ cells (Fig. 6 A). Then, the shRNA plasmids against PAC1 were further transfected into neuro2a/PACAP^-^ cells. After the data were plotted as fold changes in the cells that were transfected with control plasmids (-), it was found that PAC1 shRNA plasmids (+) significantly down-regulated the expression of PAC1 (Fig. 6 A), in turn decreasing the remaining cell viabilities to almost a half of the remaining cell viabilities of the cells that were transfected with control plasmids (-) 48 h after serum withdrawal (Fig. 6 B, P<0.01, shRNA+ vs. shRNA-). Furthermore, after the relative protein levels were normalized by the corresponding levels of the control nucleoporin-p62 and plotted as fold changes in the cells that were transfected with the control plasmids (-), the transfection with PAC1 shRNA plasmids (+) also decreased the levels of β-catenin, cyclin D1 and c-myc, significantly corresponding to the Wnt/β-catenin pathway compared to transfection with the control plasmids (-) (Fig. 6 C, P<0.01, shRNA+ vs. shRNA-). These results indicate that under ligand-free conditions, the down-regulation of PAC1 in natural cells, such as neuro2a, inhibited the anti-apoptotic activity against serum withdrawal. Therefore, it was hypothesized that the down-regulation of PAC1 promotes apoptosis in cells with a naturally high expression of PAC1, such as gliomas and medulloblastomas.

### The endocytosis of PAC1 dimers during serum-withdrawal-induced apoptosis

**Figure 7:**
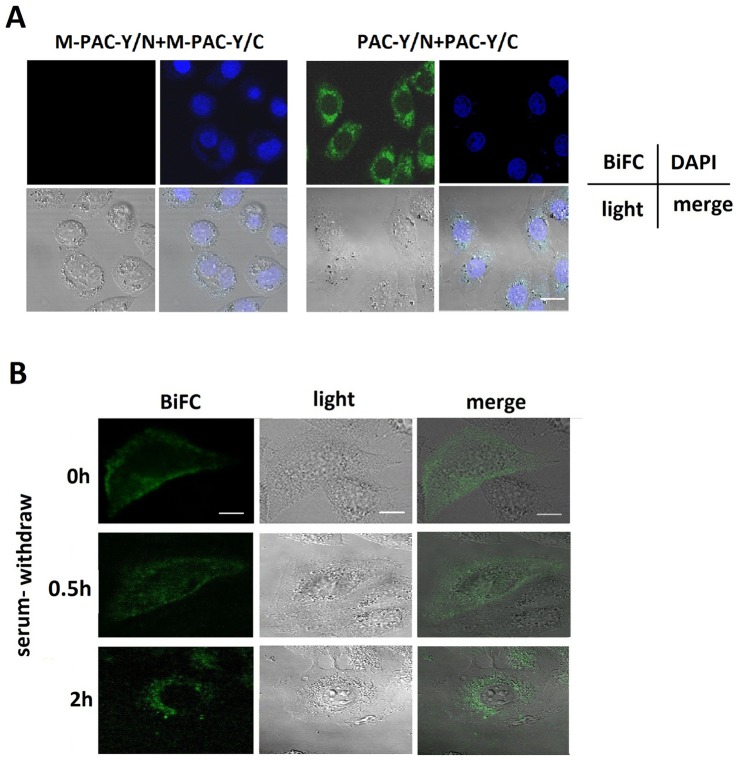
The endocytosis of PAC1 dimers using BiFC during serum withdrawal. (A) The fluorescence confocal microscopic images of BiFC signals 2 h after serum withdrawal. It was shown that after the nuclear staining with DAPI in CHO cells transfected with PAC-Y/N+PAC-Y/C, the BiFC signals (YFP fluorescence reproduced by the dimerization), representing the PAC1 dimers, were mostly located into the cells and close to the nucleus 2 h after serum withdrawal, while CHO cells transfected with M-PAC-Y/N+M-PAC-Y/C displayed no BiFC signals. Bar, 10 µm. (B) The fluorescence confocal microscopic images of live CHO cells transfected with PAC-Y/N+PAC-Y/C submitted to serum withdrawal. At the beginning of the serum withdrawal, the BiFC signals, representing the PAC1 dimers, were mostly located on or near plasma membranes. Then, at 0.5 h after serum withdrawal, the BiFC signals trafficked into the cells in some type of vehicles, and at 2 h after serum withdrawal, most of the BiFC signals were inside the cells and aggregated around the nucleus. Bar, 5 µm.

To observe the trafficking of PAC1 dimers during serum withdrawal, BiFC combined with fluorescence confocal microscopy was used to visualize the translocation of the PAC1 dimers. At 2 h after serum withdrawal, the CHO cells that were transfected with PAC-Y/N+PAC-Y/C displayed BiFC signals (YFP fluorescence reproduced by PAC1 dimerization) that aggregated inside of the cells and around the nucleus, whereas the CHO cells that were transfected with M-PAC-Y/N+M-PAC-Y/C did not produce BiFC signals because M-PAC1 failed to form dimers (Fig. 7 A). When the images were collected from 0–2 h after serum withdrawal, as shown in Fig. 7 B, the BiFC signals representing the dimers of PAC1 were mostly located on or near the membranes at the beginning of the serum withdrawal; then, the BiFC signals gradually left the plasma membrane and were internalized during serum withdrawal. Finally, the BiFC signals, representing the PAC1 dimers, were mostly located around the nucleus at 2 h after serum withdrawal. The PAC1 dimers were internalized in some type of vesicle. These images suggest that the endocytosis of PAC1 in the dimer form was associated with the ligand-independent basal activity of PAC1. Because the internalization is considered obligatory for the activation of GPCRs, the endocytosis of PAC1 dimers could be a marker for the activation of its basal activity.

## Discussion

This research revealed, for the first time, the overexpression of wild-type PAC1 in the absence of the ligand-endowed CHO cells with receptor-level-dependent activity against serum-withdrawal-induced apoptosis. In contrast, the N-terminal first Cys/Ala mutant M-PAC1, which cannot form dimers, did not confer cells with this type of ligand-independent activity against serum-withdrawal-induced apoptosis. Furthermore, the higher expression levels of PAC1 in CHO cells resulted in a higher cellular anti-apoptotic activity in a ligand-independent manner. NAC, as the inhibitor of the dimerization of PAC1, significantly inhibited this type of ligand-independent activity of PAC1. These findings suggest that PAC1 has dimer-dependent basal activity in a ligand-independent manner.

The dimerization of GPCRs affects cellular signaling by GPCRs [19]–[22]. It was shown in this study that the dimerization of PAC1 was essential for its basal activity because the N-terminal first Cys/Ala mutant M-PAC1, which cannot form dimers, did not display basal activity and that the basal activity of PAC1 could be inhibited by the inhibitor of its dimerization. Similar findings on the dimer-dependent basal activity of GPCRs have been reported, such as the dimerization of the Xenopus GPCR Frizzled-3, which is sufficient to activate the Wnt/β-catenin pathway [15]. Another example is the dimeric GABA_B_ GPCR, which locks itself in an active state [23]. The mechanism of the dimerization of GPCRs, which influences their conformation and their reactions with certain special signaling molecules, leading to the production of the ligand-independent basal activity of GPCRs, is understandable. Furthermore, the dimerization of GPCRs also influences the binding and activation of GPCR agonists, which likely is the reason why the PAC1 dimers had significantly lower reaction levels against PACAP than did the M-PAC1 monomer in this research.

Until this study, there have been no reports of the ligand-independent intrinsic/basal activity of PAC1. Although constitutive receptor activation conferred by a mutation in the second intracellular loop of PAC1 has been reported [24], this phenomenon is not equivalent to the ligand-independent basal activity that was revealed in this study. The ligand-independent basal activity of GPCRs is accepted and is considered part of the multifaceted functionality of GPCRs [13]. Many GPCRs have basal activity, which is often associated with the basal functions of the body. For example, the histamine H3 receptor shows a high level of intrinsic activity both in vitro and in vivo and regulates histamine neurons [25]. G protein-coupled opioid receptors also exhibit spontaneous activity, which is limited by ligand activation [26]. The finding of the ligand-independent basal activity of PAC1 will help us to understand the physiological and pathological roles of PAC1. PAC1 is abundant in nerves and in the neuroendocrine system and always acts as a reactive receptor against potentially damaging external factors. It was showed that the expression of PAC1 increases in the degenerative thymus [9], in the bed nucleus of the stria terminalis subjected to chronic stress [10] and in aged rat brain [7], which indicated that the PAC1 high expression may trigger a self-protective mechanism against injury and degeneration. The receptor-level-dependent basal activity of PAC1, which endows cells with anti-apoptotic activity, may help to explain the function of the elevated expression of PAC1 that is associated with nerve injury [27] or tumors [6] because elevated levels of PAC1 dimers may be responsible to produce anti-apoptotic activity against injury or promote the rapid proliferation of tumors.

We considered that the signal pathway that is produced by the dimerization of PAC1 is not the same as the signal pathway that is induced by the ligand-dependent activation of PAC1, which induced the de-dimerization of PAC1 on the plasma membrane [28], while the internalization of PAC1 dimers was associated with the dimer-dependent basal activity in this study. Furthermore, the mechanism by which the overexpression of PAC1 dimers produces ligand-independent cell signaling in tumors may also help explain the inhibitory effect of the PAC1 agonist PACAP on the proliferation of tumor cells, such as medulloblastomas [29] and serum-starved glioma cells [30], because the binding of the ligand PACAP may interrupt the dimerization and block the dimer-dependent ligand-independent cell signaling. In serum-starved glioma cells, PACAP treatment decreased the cyclin D1 levels [30]. However, in the serum-withdrawal-induced apoptosis that was described in this study, the increased PAC1 dimer levels produced higher cyclin D1 and c-myc levels, which corresponded to higher viability in the remaining cells. Therefore, in our opinion, the expression level of PAC1 dimers, which produce cell signals in a ligand-independent manner, should be considered a factor that regulates and affects the cell signals and function of PACAP. Furthermore, the finding in this study that the knockdown of PAC1 with shRNA in neuro2a promoted apoptosis suggests that the basal activity of PAC1 may be a new drug target for tumors. The effect of the PAC1 dimerization inhibitor NAC on the basal activity of PAC1 also hinted that the regulation of the dimerization of PAC1 maybe a novel way for drug development using PAC1 as a target.

In this study, the detection of β-catenin and its two targets cyclin D1 and c-myc, the determination of the effects of Wnt/β-catenin inhibitor XAV939 and the results of Top-flash assays indicated for the first time that Wnt/β-catenin pathway is involved in the dimer-dependent basal activity of PAC1. The Wnt/β-catenin pathway is the classic pathway controlling cell proliferation and apoptosis, which is closely related to cancer cells. In this study, the Wnt/β-catenin inhibitor XAV939 inhibited the anti-apoptotic activity of CHO cells with the overexpression of PAC1. A closely related report demonstrated that XAV939 promotes cell apoptosis in the neuroblastoma cell line SH-SY5Y [31]. The Wnt/β-catenin pathway involvement in PAC1 basal activity possibly contributed to the apoptotic effects of XAV939 on neuroblastoma cells, in our opinion, which deserves more attention and more-detailed research.

In this research, the significant endocytosis of PAC1 dimers during serum withdrawal was associated with the ligand-independent basal activity of PAC1. The endocytosis of receptors plays a key role in the activation and fine control of the Wnt/β-catenin signal [32],[33]. The internalization of GPCRs is considered obligatory and serves as a marker of the activation of GPCRs. Therefore in our opinion, the significant endocytosis of PAC1 dimers may be involved in the activation and transduction of the Wnt/β-catenin pathway and a marker of the activation of PAC1 basal activity. As shown in this research by fluorescence confocal microscopy, the endocytosis of PAC1 dimers was associated with certain types of vesicles, such as caveolae (Fig. 7 B). The endocytosis of PAC1 by caveolae is essential for the long-lasting and enhanced activation of PAC1 [34],[35]. In addition, caveolae are plasma membrane sensors, protectors and organizers [36]. Caveolae and/or lipid rafts not only mediate the endocytosis of GPCRs but also regulate cell signaling by GPCRs [37]. Indeed, the detailed mechanism that links the endocytosis of PAC1 dimers with the activation of its basal activity requires further research.

**Figure 8:**
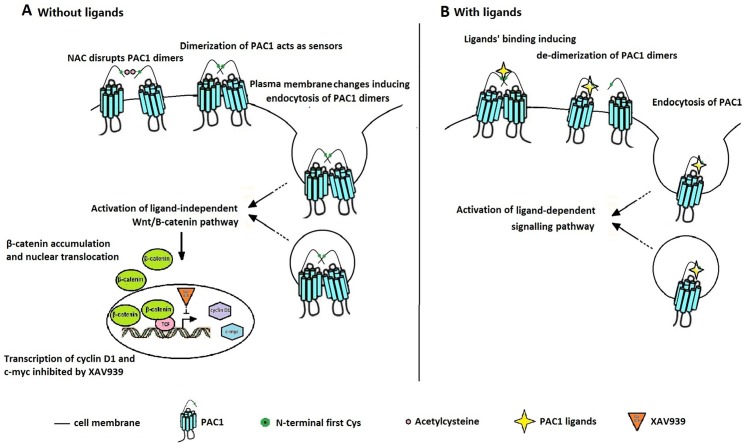
The mechanism for the ligan-independent basal activity of PAC1 dimers (A) and the ligand-dependent activation of PAC1. (A) In ligand-free situation, the disturbance of the plasma induced entocytosis of PAC1 dimers, which triggered the activation of the basal activity of PAC1 dimers involved with Wnt/β-catenin signal pathway to protect the cells against apoptosis. (B) In ligand-dependent manner, the binding of the ligands for PAC1 disrupted the dimerization of PAC1 and induced internalization of PAC1 monomer, which inhibited the basal activity of PAC1 dimers.

As shown in Fig. 8, we generated a model for the dimer-dependent, ligand-independent activation of PAC1 (Fig. 8 A) and a model for the ligand-dependent activation of PAC1 (Fig. 8 B). Under ligand-free conditions, the PAC1 dimers on the plasma membrane acted as sensors, and the changes in the plasma membrane inducing the endocytosis of PAC1 dimers triggered the basal activity of PAC1. The binding of the ligands for PAC1, such as PACAP, disrupted the dimerization of PAC1 on the plasma membrane as shown by our previous report [28]. In turn, due to steric hindrance, the dimerization of PAC1 interfered with the binding of the ligands to the PAC1 monomer, which is why the M-PAC 1 monomer was more sensitive to PACAP binding than the PAC1 dimer. The reagents that disrupted the dimerization of PAC1 inhibited the basal activity of PAC1 dimers, such as the exogenous oligopeptide HSDCIF, which interfered with the PAC1 dimers through the Cys residue, reducing the cell viabilities of PAC1-CHO [14]. However, compared to those of NAC, the effects of the oligopeptide HSDCIF with high homology with PACAP (1–5) (HSDGIF), which, corresponding to the activation of PAC1, were more complex. The exogenous oligopeptide HSDCIF not only interrupted the dimerization of PAC1 but also induced the internalization of the PAC1 monomer, which could be why the exogenous oligopeptide HSDCIF inhibited the binding and the activation of PAC1 by PACAP [14]. It is shown in Fig. 8 that both the ligand-dependent and the ligand-independent activation PAC1 were accompanied by the endocytosis of the receptor, indicating that the endocytosis of the receptor plays an important role in cell signal transduction. However, we hypothesize that the PAC1 endocytosis mechanism during ligand-independent activation may be different from that during ligand-dependent activation, which deserves more research.

In summary, the data in this research indicate that the PACAP-preferring receptor PAC1 has significant dimer-dependent intrinsic/basal activity, which is involved in Wnt/β-catenin signaling and is associated with the endocytosis of PAC1 dimers. This finding suggests that increases in the expression levels of PAC1 dimers may produce cell signals in a ligand-dependent manner to protect cells from apoptosis or to promote cell proliferation; in contrast, the knockdown of PAC1 or interference with the dimerization of PAC1 may interrupt the basal activity of PAC1 to promote cell apoptosis. Measurement of the basal activity of PAC1 and the exploration of the mechanism of the basal activity of PAC1 will not only help us understand the physiological and pathological functional roles of PAC1 but will also help the development of drugs targeting PAC1. For example, molecules that interrupt the dimerization of PAC1 or that block the endocytosis of PAC1 dimers may inhibit the basal activity of PAC1, which may have potent effects against some tumors or nerve diseases that are associated with PAC1.
